# Stress Evaluation by Hemoglobin Concentration Change Using Mobile NIRS

**DOI:** 10.3390/brainsci12040488

**Published:** 2022-04-11

**Authors:** Shingo Takahashi, Noriko Sakurai, Satoshi Kasai, Naoki Kodama

**Affiliations:** 1 Department of Healthcare Informatics, Faculty of Health and Welfare, Takasaki University of Health and Welfare, 37-1 Nakaorui-machi, Takasaki 370-0033, Japan; takahashi-shin@takasaki-u.ac.jp; 2 Department of Radiological Technology, Faculty of Medical Technology, Niigata University of Health and Welfare, 1398 Shimami-cho, Kita-ku, Niigata 950-3198, Japan; noriko-sakurai@nuhw.ac.jp (N.S.); satoshi-kasai@nuhw.ac.jp (S.K.)

**Keywords:** near-infrared spectroscopy, stress, trail-making test, prefrontal cortex, laterality index

## Abstract

Previous studies have reported a relationship between stress and brain activity, and stress has been quantitatively evaluated using near-infrared spectroscopy (NIRS). In the present study, we examined whether a relationship exists between salivary amylase levels and brain activity during the trail-making test (TMT) using mobile NIRS. This study aimed to assess stress levels by using mobile NIRS. Salivary amylase was measured with a salivary amylase monitor, and hemoglobin concentration was measured using Neu’s HOT-2000. Measurements were taken four times for each subject, and the values at each measurement were evaluated. Changes in the values at the first–second, second–third, and third–fourth measurements were also analyzed. Results showed that the value of the fluctuations has a higher correlation than the comparison of point values. These results suggest that the accuracy of stress assessment by NIRS can be improved by using variability and time-series data compared with stress assessment using NIRS at a single time point.

## 1. Introduction

Stress has several effects on the human body and is an important health factor. High levels of stress can cause a variety of symptoms, including digestive disorders such as stomach pain, obesity due to appetite and weight gain, lowered immunity, nervous system disorders such as anxiety, depression, and sleep disorders, and cardiovascular disorders such as increased blood pressure, heart rate, and blood fats [1]. Stress assessment and reduction are necessary because people who work or live in stressful environments are more likely to suffer from many disorders [2].

Stress levels can be evaluated by the measurement of biomarkers. Potential markers of stress include thermal stress markers, such as heat shock proteins (HSPs), innate immune markers, such as Acute Phase Proteins (APPs), oxidative stress markers, and chemical secretions in the saliva and urine [3]. In recent years salivary alpha-amylase has emerged as a valid and reliable marker of autonomic nervous system activity in stress research [4]. Various studies have been conducted to assess stress, and simple approaches to measuring stress using devices have been developed. A study has identified facial features obtained from eye, mouth, head, and heart movements by using a camera as suitable indicators of stress and anxiety [5]. Heart rate variability is also used as an indicator of psychological stress [6]. Quantitative assessments of stress have been widely conducted. Finger-point volumetric pulse wave (FPG) has been used to quantitatively assess psychological stress [7].

A comparison of the performance of a near-infrared spectroscopy (NIRS) device and conventional methods, such as electroencephalography and peripheral arterial tonometry, showed that total hemoglobin levels increase in the frontal cortex during stress tasks [8]. A study reporting that the prefrontal cortex (PFC) plays an important role in stress response suggests that mental stress tasks activate the bilateral PFC [9], suggesting that stress and brain activity are related. In a study aimed at determining the active areas of brain function and electroencephalography features in stress accumulation, left–right differences in brain activity measured using NIRS are confirmed whenever stress accumulates [10], indicating that a NIRS device can be used to evaluate stress. In recent years, devices measuring brain activity using near-infrared light have become smaller. Consequently, mobile devices, which are expected to have various applications, including measurement of brain activity, have been developed.

In this study, we aimed to measure the brain activity of healthy young people using HOT-2000, a mobile brain activity measurement device, and examine the relationship between brain activity and stress. Measurements were performed four times a month. The correlation between salivary amylase levels and brain activity at each measurement, as well as the relationship between salivary amylase levels and fluctuations in the measurements, were also investigated.

## 2. Materials and Methods

### 2.1. Subjects and Methods

**Figure 1:**
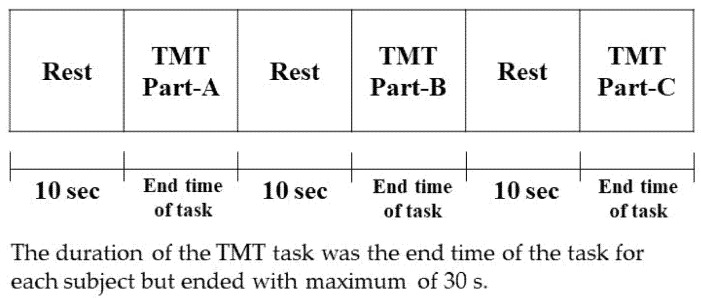
Measurement protocol.

Subject information for each measurement is presented in Table 1. A total of 20 subjects (8 men, 13 women, age 20.7 ± 0.5 years) were included. Salivary amylase level Trail Making Test-A (TMT-A), Trail-Making Test-B (TMT-B), and Trail-Making Test-C (TMT-C) completion times and hemoglobin concentration were measured. The hemoglobin concentration was measured during the TMT task. Measurements were taken four times for each subject, and the values at each measurement were evaluated. Changes in the values at the first–second, second–third, and third–fourth measurements were also analyzed. Each measurement was conducted monthly. Salivary amylase was measured using a salivary amylase monitor, and hemoglobin concentration was measured using HOT-2000 (Neu). TMT-A, TMT-B, and TMT-C were assessed in an experimental block design, with all blocks separated by a 10-s rest period. The duration of the TMT task was the end time of the task for each subject; however, the task ended after a maximum duration of 30 s. The experimental protocol is illustrated in Figure 1.

The right side of the PFC has been suggested to be important in the regulation of the stress system, and the laterality index (LI) is an indicator of stress in the left and right differences in the PFC [11,12]. Studies found an asymmetrical relationship between stress and NIRS. In the present study, we defined LI ((R − L)/(R + L)) as the dominance of the left and right prefrontal cortices [11,13]. R was defined as the mean value during the task on the right side, and L was defined as the mean value during the task on the left side. The right side of the PFC is important in the regulation of the stress system [12]. Statistical analyses were performed using SPSS Statistics 27.0 by IBM (Chicago, Illinois, the United States of America). SPSS Statistics 27.0 (IBM, Chicago, Illinois, the United States of America) is a statistical software platform. Pearson’s correlation coefficient was calculated to determine the correlation between the amount of change and the task. Multiple comparisons were corrected by Bonferroni correction. The significance level was set at 5%.

Prior to study initiation, the study contents, measurement details, equipment safety, and expected results were explained to all subjects. Written consent was also obtained from the subjects. This study was approved by the Research Ethics Committee of the Niigata University of Health and Welfare (Approval no.: 18518-201111).

### 2.2. TMT

The TMT consists of parts A (TMT-A) and B (TMT-B). In the TMT-A, the subject is instructed to draw a line connecting 25 numbers on a sheet of paper in ascending order. The TMT is measured according to completion time and the number of errors. The difference between the completion times for parts A and B (part B–part A) and the ratio is also determined (B/A) [14]. The TMT requires visual exploration, scanning, processing speed, mental flexibility, and executive functioning [15].

**Figure 2:**
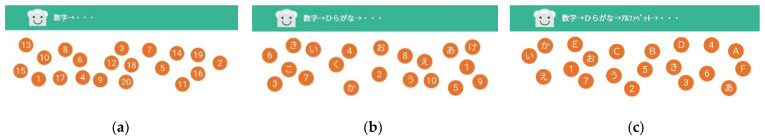
TMT of tablet manufactured by NeU using in the study. (**a**) TMT-A, (**b**) TMT-B, (**c**) TMT-C. TMT-A is task to draw a line connect numbers. The TMT-B, which includes a task to connect numbers and letters of Japanese (hiragana), and TMT-C, which includes a task to connect numbers, hiragana, and alphabets in order, were also conducted.

The TMT is not only an indicator of cognitive decline but is also related to general intelligence [16]. In recent years, devices have been developed to perform the TMT electronically [17]. In this study, the TMT was performed using a tablet manufactured by NeU (Chiyoda-Ku, Tokyo, Japan). The tablet allows TMT-A, TMT-B, and TMT-C to be performed continuously, with a maximum task duration of 30 s and a time setting of 10 s for waiting between tasks. In addition to the TMT-A, the TMT-B, which includes a task to connect numbers and letters of Japanese (hiragana), and TMT-C, which includes a task to connect numbers, hiragana, and alphabets in order, were also conducted (Figure 2).

### 2.3. HOT-2000

**Figure 3:**
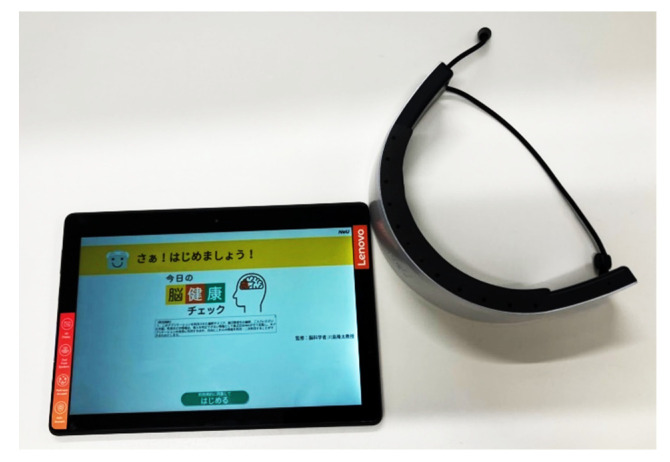
Measurement device. Figure 3 shows the HOT-2000 and the tablet that performed the TMT (for Japanese).

**Figure 4:**
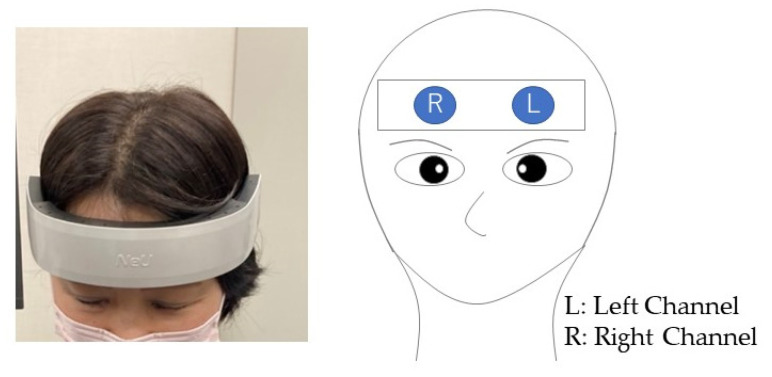
Probe set in in the frontal lobe.

HOT-2000 is a two-channel wearable portable brain activity measurement device that uses near-infrared light to monitor blood flow changes in the frontal lobe (Figure 3). Figure 4 shows the HOT-2000 with the probe set. HOT-2000 uses a laser beam with a wavelength of 810 nm and a sampling rate of 10 Hz. It thereby makes it possible to measure brain activity by detecting total blood flow. In this study, the raw data of signals obtained from the HOT-2000 were analyzed. Brain activity during the task was defined as the mean value of the signal during the task minus the mean value at rest immediately before the task (change in hemoglobin). The device was worn such that the headset was close to the forehead and above the eyebrows. Cotton with alcohol was used to wipe off sweat or dirt on the forehead.

The HOT-1000, an older version of the HOT-2000, has been used in various research projects, including psychophysical evaluation of driver psychological states in autonomous vehicles [18]. It has also been reported that HOT-1000 correlates with TNIRS [19].

### 2.4. Salivary Amylase Monitor

**Figure 5:**
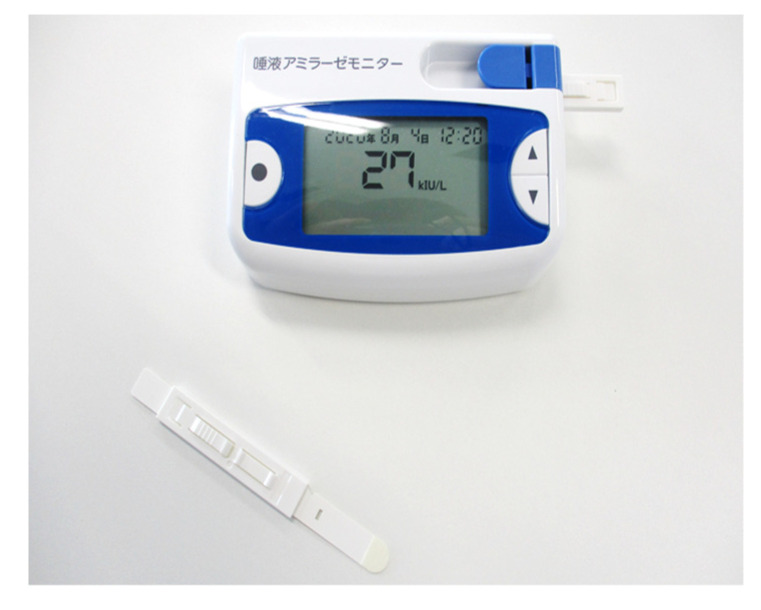
Salivary amylase activity monitor (for Japanese).

A salivary amylase monitor manufactured by Nipro Corporation (Osaka, Japan) was used to evaluate stress (Figure 5). This device consisting of the main unit and a chip, is designed to collect saliva and measure amylase contained in saliva.

Salivary alpha-amylase has been shown to respond more quickly to stress [20] and has been reported to be useful for stress assessment because it correlates with autonomic function (low frequency–high frequency) [21].

## 3. Results

**Figure 6:**
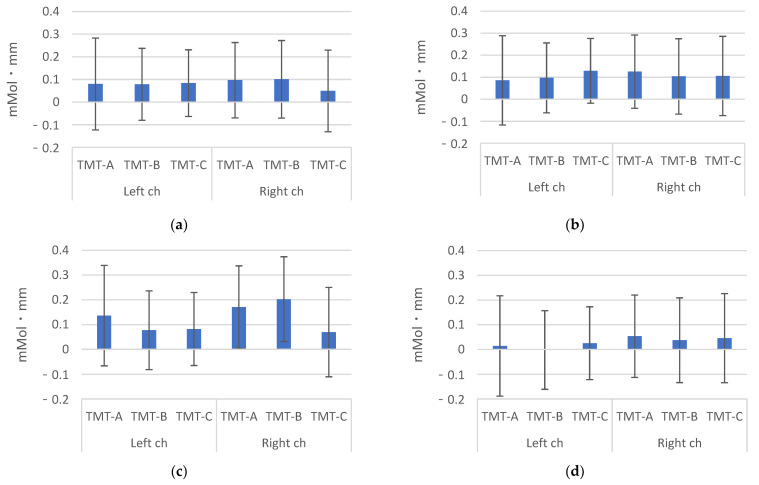
Data of HOT-2000. (**a**) first time, (**b**) second time, (**c**) third time, (**d**) fourth time.

**Figure 7:**
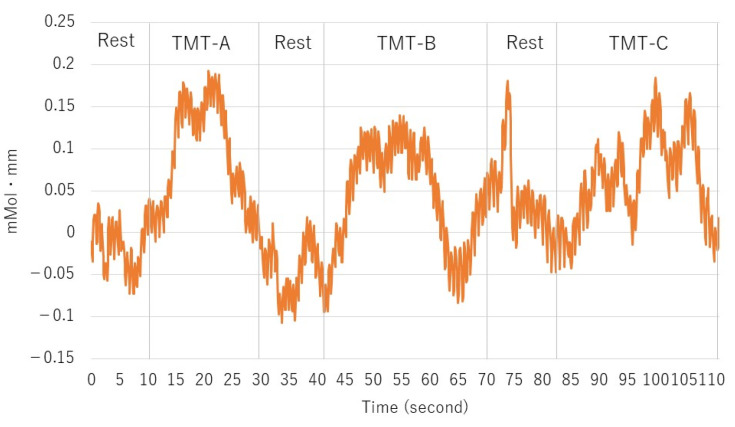
One example of a curve for the signal.

The subject information, salivary amylase concentration, and TMT results are shown in Table 1. The hemoglobin concentrations (Change in hemoglobin) measured using HOT-2000 are shown in Figure 6. In the first measurement, the TMT-C value was lower in the right channel. In the fourth measurement, the change was smaller compared with the other results. One example of a curve for the signal by HOT-2000 is illustrated in Figure 7.

The correlation coefficients between the hemoglobin concentrations (Change in hemoglobin) measured using HOT-2000 and the salivary amylase values are shown in Table 2. No correlations were found for any of the items in the first, third, or fourth measurements. Table 3 shows the correlation coefficients between the difference in hemoglobin concentrations measured using HOT-2000 and the salivary amylase values between the first and second, second and third, and third and fourth measurements. The first and second measurements showed no correlation between hemoglobin concentrations fluctuations and fluctuations in salivary amylase values. The second and third measurements showed a correlation between fluctuations in the right and left channels of the TMT-C and fluctuations in salivary amylase values. In the third and fourth measurements, a significant correlation was found between the variation in the left side channel of TMT-C and the variation in salivary amylase value. The correlation between salivary amylase values in LI and between the difference in LI and salivary amylase values are shown in Table 4. In LI, the correlation was not as strong as for the hemoglobin concentration.

**Figure 8:**
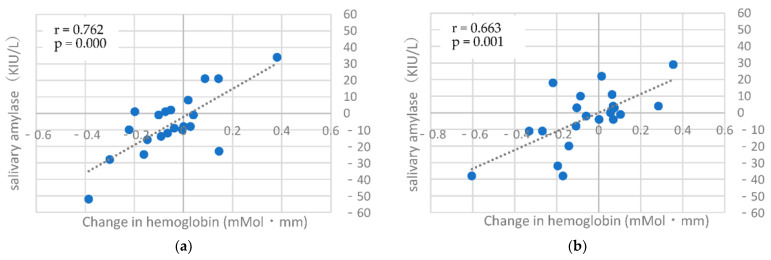
Correlation diagram of the difference in hemoglobin concentration change (TMT-C Left) and the difference in amylase value. (**a**) The second and third measurements fluctuations, (**b**) The third and fourth measurements fluctuations.

Figure 8 shows the 2–3 and 3–4 correlograms of TMT-C (Left), where the correlation was strong.

## 4. Discussion

Two types of TMTs are currently used as functional tests: parts A and B. Part B not only measures a more difficult cognitive task but also places greater demands on motor speed and visual search, which are known to result in lower performance compared with part A [22]. In the present study, the time required to complete a task in part B is longer than that in part A, which is similar to previous findings. In the present experiment, all subjects performed part A first, followed by part B and then part C. In part C, the difficulty of the task was further increased by the use of alphabets in addition to numbers and hiragana, which may have reduced the performance. Because the raw NIRS data are relative, they cannot be directly compared among subjects or channels [23,24]. Therefore, performing a comparison by each channel or number of measurements is not possible. However, the comparison for each TMT showed lower values for the TMT-C and higher values for the TMT-A and TMT-B. When the task is too easy or difficult, no increase has been reported in cerebral blood flow. The TMT-C was a difficult task, which may have resulted in lower values.

With regard to the measurement of brain activity using near-infrared light, it has been reported that a relationship exists between the activity of the PFC and stress and that NIRS is useful for stress assessment. Previous studies have reported that left and right asymmetric activation in the PFC is useful as an assessment, and others have reported that brain activity on the right side is related to stress [25]. In the present study, significant correlations were found in the TMT-C of the right side for the first and second sessions, in the TMT-C of the bilateral channels for the second and third sessions, and in the TMT-C of the left side channel for the third and fourth sessions. These results suggest that mobile NIRS can be used to assess stress. However, the hemoglobin concentrations measured using HOT-2000 and salivary amylase values at a single time point (Table 2), rather than variability, showed lower correlation coefficients than the variability results. NIRS shows large individual differences, and the values of salivary amylase were used for stress assessment in this study. The amount and activity of salivary amylase vary among individuals [26]. Measurement performed at one time point is considered to be weakly related to stress. Therefore, the relationship between NIRS and salivary amylase varies greatly among individuals, and evaluating stress by measuring frontal lobe activity at a single time point using NIRS is difficult. This measurement was obtained from changes measured every month. Therefore, it is necessary to examine different measurement periods in the future, such as first and third, first and fourth, etc.

The three limitations of this study are as follows. First, stress was measured only using salivary amylase. There are several stress hormones; however, amylase responds quickly to stress. Second, only TMT was used. Third, the rest period for NIRS measurements was short. In this study, the rest period was 10 s because the TMT was performed on a tablet. Therefore, it was not adequate to precisely evaluate brain activity for each task. Since this study investigated the relationship between portable NIRS measurements and stress, future studies on each task and stress are warranted. Additionally, no baseline correction or normalization was performed on the NIRS signal because we were investigating the relationship between the NIRS signal and stress. In the future, correction should also be performed for the mobile NIRS signal and also for indices more strongly associated with stress.

## 5. Conclusions

The correlation coefficient between the difference in salivary amylase values and brain activities by variation was higher than that between the salivary amylase values and brain activity in each measurement. This suggests that the accuracy of stress assessment by NIRS can be improved by using variability and time-series data, compared with stress assessment by NIRS measurement at a single time point.

The results of this research will enable early detection of stress-induced mental disorders and easy stress assessment in companies.

## Figures and Tables

**Table 1:** Subject information.

	Salivary Amylase (KIU/L)	TMT-A (Time)	TMT-B (Time)	TMT-C (Time)
First measurement	17.2 ± 9.1	16.7 ± 3.3	24.2 ± 5.4	29.9 ± 1.2
Second measurement	23.9 ± 12.8	15.9 ± 2.1	23.2 ± 4.1	29.0 ± 2.2
Third measurement	17.8 ± 14.3	16.0 ± 2.1	22.8 ± 4.2	27.2 ± 3.6
Fourth measurement	15.4 ± 13.8	16.4 ± 2.5	23.7 ± 7.2	28.0 ± 3.0

**Table 2:** Correlation between changes in hemoglobin concentration and amylase levels.

	TMT-A Left	TMT-B Left	TMT-C Left	TMT-A Right	TMT-B Right	TMT-C Right
First measurement	0.063	0.424	0.256	0.063	0.277	0.148
Second measurement	0.358	0.315	0.417	0.342	0.373	0.613 *
Third measurement	0.031	−0.159	0.226	−0.191	−0.416	0.123
Fourth measurement	0.295	0.123	0.374	−0.044	−0.182	0.143

* p < 0.05.

**Table 3:** Correlation between the difference in hemoglobin concentration change and the difference in amylase value.

	TMT-A Left	TMT-B Left	TMT-C Left	TMT-A Right	TMT-B Right	TMT-C Right
1–2	−0.304	−0.157	0.188	0.053	0.039	0.486
2–3	−0.006	0.265	0.762 **	−0.032	−0.189	0.602 **
3–4	0.252	0.126	0.663 **	0.014	−0.310	0.242

** *p* < 0.01.

**Table 4:** Correlation between LI and LI variability and salivary amylase levels.

	LI (First)	LI (Second)	LI (Third)	LI (Fourth)	LI (1–2)	LI (2–3)	LI (3–4)
TMT-A	0.109	0.539 *	0.059	0.032	0.469	0.637 *	0.134
TMT-B	0.090	0.172	−0.347	0.117	0.140	0.349	−0.074
TMT-C	0.108	0.142	−0.133	−0.036	0.075	−0.079	0.069

* p < 0.05.

## Data Availability

The data sets used and analyzed in the current study are available from the corresponding author upon reasonable request.
